# Revelation of the mediation role of moral sensitivity on safety attitude and personality traits among critical care nurses

**DOI:** 10.1186/s12912-025-02868-6

**Published:** 2025-03-08

**Authors:** Hind Ismail Ali, Ahmed Abdellah Othman, Mohamed Hussein Ramadan Atta, Sanaa Saber Mohamed, Seham Hassan Mohamed

**Affiliations:** 1 Nursing Administration, Faculty of Nursing, Sohag University, Sohag City, Egypt; 2 Psychiatric and Mental Health Nursing Department, Faculty of Nursing, Alexandria University, Alexandria, Egypt; 3 Critical Care and Emergency Nursing, Faculty of Nursing, Sohag University, Sohag City, Egypt

**Keywords:** Critical care nurses, Mediation role, Moral sensitivity, Personality traits& safety attitude

## Abstract

**Background:**

Critical care nurses face complex ethical dilemmas and high-pressure situations that require quick ethical decision-making. Personality traits have been recognized as influencing individuals’ ethical decision-making processes and attitudes toward safety in healthcare. Moral sensitivity helps nurses recognize ethical issues and respond appropriately to these challenges. So, this study aimed to assess the mediation role of moral sensitivity on safety attitudes and personality traits among critical care nurses.

**Method:**

This study used a convenience sample method and a descriptive correlational research design to conduct it on 232 critical care nurses who worked at intensive care units and emergency departments of nine Sohag Governmental Hospitals. Three tools were used to collect data: the Moral Sensitivity Questionnaire (MSQ), the Big Five Factors of Personality Inventory sheet, and the Safety Attitudes Questionnaire (SAQ). The data were analyzed using descriptive statistics and inferential tests (multivariate linear regression using the backward method).

**Result:**

The study revealed the personality profiles of the participants, with a total personality traits mean score (150.012 ± 9.628) and higher mean scores in conscientiousness and openness. The highest mean in moral sensitivity was interpersonal orientation, 22.76 ± 3.339, and moral meaning, 26.97 ± 4.279. Participants had low average mean scores regarding safety attitude 73.254 ± 11.368. There was a positive correlation between personality traits, moral sensitivity, and safety attitude. Finally, moral sensitivity acted as a mediating factor between personality traits and safety attitude.

**Conclusion:**

The results of the present study suggest that personality assessment and moral sensitivity training be incorporated into nursing education and professional development programs. By enhancing nurses’ self-awareness and sensitivity to ethical dilemmas, institutions can potentially improve safety attitudes and, consequently, patient care outcomes.

**Supplementary Information:**

The online version contains supplementary material available at 10.1186/s12912-025-02868-6.

## Introduction

Every individual is inherently entitled to the right to health, ensuring access to affordable, secure, and high-quality healthcare devoid of any impediments based on geographic location or healthcare providers [1]. Healthcare organizations now place a high premium on patient safety, which presents a challenge to hospital management. Nurses encompass a multifaceted construct, encapsulating their collective beliefs, perceptions, and values regarding patient safety and the surrounding work environment. This paradigm reflects the approach of nurses towards safety practices, ranging from positive safety-conscious attitudes to attitudes that may be less conducive to maintaining a safe environment [2].

Nurses’ safety attitudes impact the quality of patient care and the overall health of healthcare environments [3]. A positive safety attitude cultivates a sense of accountability and responsibility among nurses, engendering a collaborative and supportive atmosphere conducive to optimal patient outcomes. It is imperative to recognize and comprehend the influential factors shaping safety attitudes among nurses [4, 5].

Nurses’ ethical values and personalities are the most essential factors affecting nurses’ safety attitudes. Disparities in personality traits, such as conscientiousness or risk aversion, shape how nurses perceive and prioritize safety measures. Additionally, nurses’ moral values and ethical considerations influence commitment to patient safety [6].

Personality is the distinctive set of patterns influencing an individual’s actions, behavior, motivations, and emotions across various contexts and plays a crucial role in shaping moral decision-making. The Big Five Personality Traits Model categorizes these traits as Neuroticism, Extraversion, Openness, Agreeableness, and Conscientiousness [9–10]. According to Abbasi-Asl, Naderi, and Akbari (2016) [11], these personality traits play a crucial role in evaluating moral foundations such as authority, purity/sanctity, and loyalty.

Moral sensitivity denotes the capacity to discern patient vulnerabilities, anticipate the ethical implications of challenging decisions, and enable nurses to deliver more fitting and ethically sound care. Moral sensitivity augments proficiency in resolving conflicts arising from differing values. As highlighted by Esmaelzadeh et al. in 2017 [3], nurses endowed with refined empathetic skills tend to excel in moral sensitivity (Türkmen et al., 2023) [12].

The development of moral awareness, sensitivity, and attitudes typically occurs during nursing education, as indicated by studies such as Shayestehfard et al. in 2020 and Lim, Park, and Shin in 2017 [7–8]. Overall, moral sensitivity empowers nurses to recognize moral dilemmas, intuitively understand the vulnerable position of patients in different situations, and comprehend the ethical ramifications of healthcare decisions, correlating with their personality traits that contribute to safety attitudes in nursing practice (Darzi-Ramandi et al., 2023) [13].

Moral judgment, when faced with ethical dilemmas, can be influenced by differences in personality traits [14]. Recent research has delved into the connections between fundamental personality traits and various factors that impact moral judgments [14–15]. Our study uses the CNI model as a framework to quantify an individual’s characteristics and moral norms. This model includes three parameters: consequence sensitivity (C), norm sensitivity (N), and generalized action versus inaction preferences (I). The CNI model, when applied to moral dilemmas, has shown systematic relationships between sensitivity to moral norms and personality traits as measured by the Personality Inventory and Big Five trait models [16–17].

Critical care nurses, in particular, are likely to confront moral challenges, making moral sensitivity crucial in their decision-making processes [18]. Critically ill patients confront the peril of injuries, fatalities, or disabilities due to the reception of substandard medical care, with a staggering 2.6 million patient deaths reported because of failing to meet safety criteria [19]. Consequently, there is an escalating imperative for nurses to exhibit adeptness in making ethical decisions and to prioritize the ethical dimensions of patient care, necessitating a heightened awareness of moral issues inherent to their professional domain [20].

The literature highlights several key findings of Mohammadi, Borhani, and Roshanzadeh in 2017 [21], which identified that critical care nurses who lack sufficient executive power for moral performance may experience moral distress, irrespective of their varying levels of moral sensitivity. Additionally, Salih et al., in 2021 [22], noted a neutral attitude among nurses toward patient safety at an Egyptian University Hospital, highlighting strong associations with educational level, years of experience, and participation in training courses on patient safety. Despite these insights, there remains a notable gap in the literature concerning the relationship between personality traits and moral sensitivity among critical care nurses in shaping their attitudes toward safety.

**Fig. 1:**
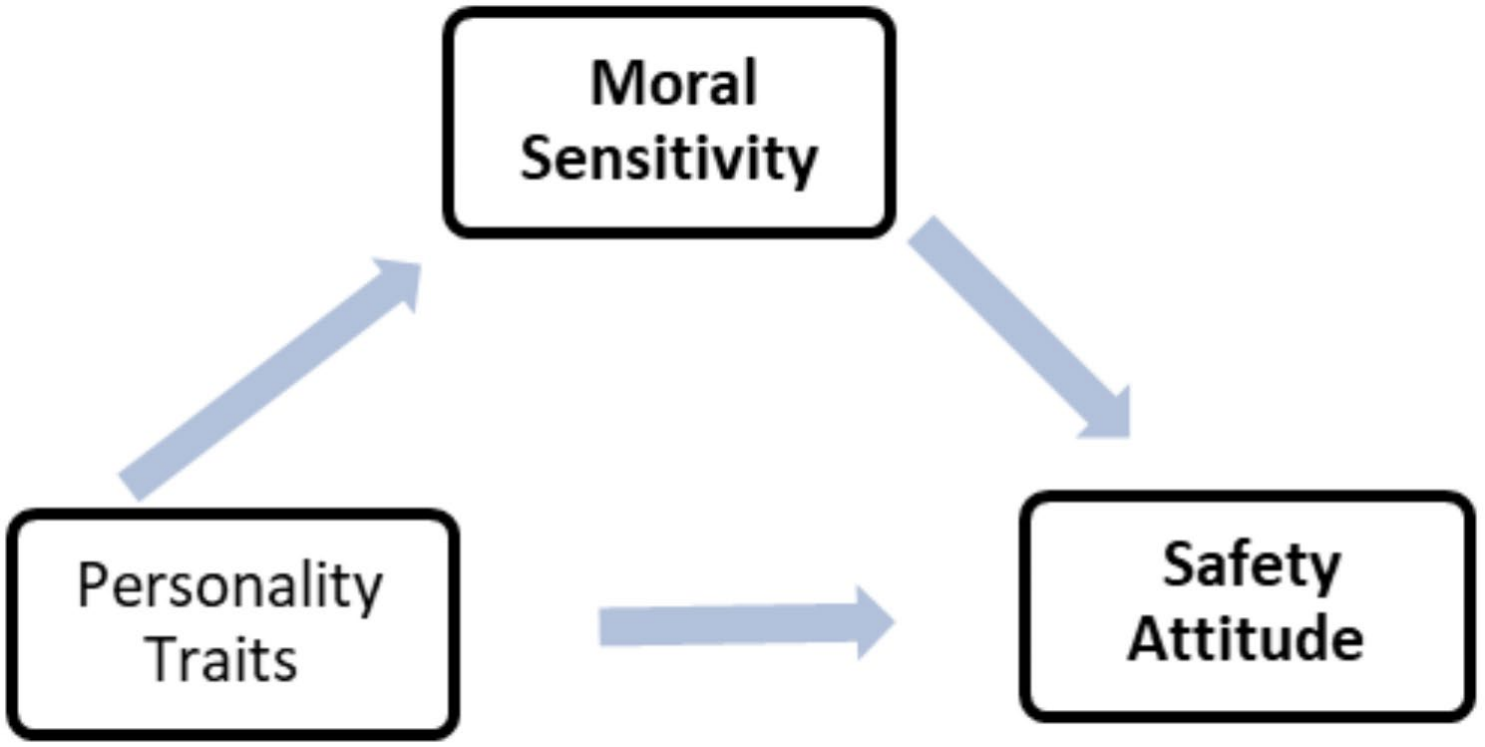
Conceptual framework model

The revealing of the role of moral sensitivity on safety attitudes and personality traits among critical care nurses offers a nuanced understanding of the intricate interplay between these variables. Through a deeper understanding of moral sensitivity’s impact, nurses will be more likely to respond to ethical dilemmas and moral distress to improve safety practices and patient outcomes. Lastly, by illuminating the factors influencing moral sensitivity, this study can provide critical support for ethical decision-making in nursing practice, equipping nurses with the necessary tools to navigate complex ethical dilemmas and uphold the highest standards of patient care. Our study has two aims: first, to assess the mediation role of moral sensitivity on personality traits and the safety attitude of critical care nurses; second, to assess the correlation type between personality traits and moral sensitivity; and third, to assess the safety attitude of critical care nurses. Two hypotheses are presented in this study (Fig. 1).

### Hypothesis 1

Moral sensitivity has a mediating role in personality traits and safety attitudes among critical care nurses.

### Hypothesis 2

Personality traits have a positive relation with moral sensitivity and safety attitude among critical care nurses.

### Operational definition

Critical care nurses provide care for critically ill patients. They are responsible for all patient care, from medication administration to tracheotomy and other ventilator care. They are equipped with the skills necessary to perform Cardiopulmonary resuscitation (CPR) and other life-saving measures at all times (Franjic, 2020) [23]. This study is about critical care nurses who deliver complex critical care in intensive care units (ICUs) and emergency departments (EDs).

## Methods

### Research design & setting

A descriptive correlational research design was used to conduct this study at 24 critical care units (15 intensive care units and 9 emergency departments) of nine Sohag Governorate hospitals. The nine Governorate hospitals were considered the main hospitals in Sohag City and had a reasonable number of critical care nurses who worked in intensive care units and emergency departments. Those governmental hospitals affiliated with the Sohag Health Directorate provide specialized medical services for a large number of populations at the governorate level. The capacity of beds at each hospital ranged from 150 to 200 beds.

### Participants and sample size calculation

A convenient sample of all critical care nurses working in the selected setting. The sample size was determined based on Open Epi, Version 3, which is an open-source calculator. The total population size of critical care nurses in the selected setting was 1504.

The parameters used in the calculation were as follows: a hypothesized percentage frequency of the outcome factor in the population (p) of 50% with a ± 5% margin of error, a confidence level of 95%, a design effect (DEFF) of 1 (applicable for cluster surveys), and the formula for sample size calculation: sample size (n) = [DEFF * N * p * (1-p)] / [(d^2 / Z^2) * (N-1) + p * (1-p)]. Based on these parameters, the minimum required sample size was 232.

The eligibility criteria for critical care nurses are currently employed as a critical care nurse in an intensive care unit or emergency department; having a minimum of one year of experience in this role; being available to complete the required questionnaires, having a proficient understanding of the language of administered questionnaires, and not having a history of mental health disorders, or other conditions that may impede participation or response accuracy.

### Study tools

Four main tools were used to collect data in the current study after reviewing the related and current literature as follows:

#### Tool one: personal data sheet

It included data about age, gender, marital status, department, years of experience, qualifications, current position, and previous training/courses about safety attitude (Supplementary Material 1).

#### Tool two: moral sensitivity questionnaire (MSQ)

Han et al. adopted it in 2010 [24]. The MSQ is used to assess moral sensitivity levels among nurses. It includes 27 items subdivided into six subscales: modifying autonomy (7 items), reliance on medical authority (4 items), moral meaning (5 items), expressing benevolence (4 items), experiencing conflict (3 items), and interpersonal orientation (4 items).

The nurses’ responses were measured on a. A 7-point response scale (from 1‘not at all’ to 7 ‘strongly agree’) was used to explore the extent of agreement with moral sensitivity. Higher scores indicated a greater degree of moral sensitivity. It is a valid and reliable tool for assessing moral sensitivity among individuals, supported by strong construct validity, divergent validity, convergent validity, and high Cronbach’s Alpha values of 0.76. In our current study, which utilized the Arabic version of the moral sensitivity questionnaire, as established by Bayoumy, Alhalabi, and Esheaba (2017) [25].

#### Tool three: big five factors of personality inventory sheet

This inventory was adopted from John & Srivastava in 1999 [26]; the 44-item inventory measures an individual on the Big Five Factors (dimensions) of personality. Each item was scored based on a five-point Likert rating from 1 to 5 (1: strongly disagree, 2: disagree, 3: no difference, 4: agree, 5: strongly agree). We used the Arabic version of The Big Five Inventory (BFI) in our study, as established by Alansari (2016) [27]. 

The reliability coefficient (Cronbach’s Alpha) for each factor of the personality traits was 0.859 (openness to experience, 10 items), 0.888 (conscientiousness, 9 items), 0.725 (extraversion, 8 items), 0.774 (Agreeableness, 9 items), and 0.736 (neuroticism, 8 items). The reliability of the Big Five Factors of Personality traits was measured by Cronbach alpha coefficient, and it was above 0.7. Thus, the validated measures of the Big Five Inventory were deemed consistent and reliable throughout the study [28].

#### Tool four: safety attitudes questionnaire (SAQ)

t was adopted by Bondevik et al. in 2014 [29], and it was adapted into an Arabic version by Elsous and colleagues in 2017 [30] that was used by the researcher in this study to assess nurses’ perception regarding safety culture, staff perception of safety must be frequently measured. Safety Attitudes aspects were classified into six dimensions (30 items): Teamwork climate (6 items), Safety climate (7 items), Perceptions of management (4 items), job satisfaction (5 items), Working conditions (4 items), and Stress recognition (4 items). Each question was scored based on a five-point Likert rating from 1 to 5 (1: strongly disagree, 2: disagree, 3: no difference, 4: agree, 5: strongly agree).

Bondevik et al., in 2014 [29], found that the Safety Attitudes Questionnaire had a Cronbach’s alpha score of 0.70, indicating acceptable internal consistency. Their confirmatory factor analysis revealed that the six-factor model of the questionnaire, which includes Teamwork climate, Safety climate, Job satisfaction, Perceptions of management, working conditions, and Stress recognition, demonstrated a good fit in a nursing home setting. Positive scoring was used for teamwork climate, management perception, safety climate, job satisfaction, and working conditions dimensions. Stress recognition had a reverse score, so the lower the score, the better the respondent’s safety attitude.

### Procedure

#### Ethical considerations

The study obtained institutional permission from the hospitals where the research was conducted and received approval from the Ethics Committee of the Faculty of Nursing, Sohag University, with approval number 8 dated January 15, 2023. The research adhered rigorously to established ethical principles in clinical research, ensuring the utmost protection of participants’ rights and welfare.

Stringent measures are implemented to guarantee the anonymity and confidentiality of all collected data, safeguarding the privacy of participants. Before their involvement in the study, all participants provided both written and verbal consent, demonstrating their voluntary agreement to participate. It is important to note that the study posed no risk to the participants throughout the research process, underscoring the meticulous attention to ethical considerations and participant well-being.

### Data collection

Data was collected over six months, from 15 January 2023 to July 2023, using an Arabic version of a self-administered questionnaire after getting permission from the original authors. The face validity of the tool was ensured through expert review, confirming that the instruments accurately capture the intended constructs. The reliability of the tool was assessed using Cronbach’s alpha, with the MSQ demonstrating a coefficient of 0.85, indicating high internal consistency, the Big Five Factors inventory showing coefficients ranging from 0.70 to 0.80 for each personality factor, and the SAQ exhibiting coefficients ranging from 0.75 to 0.85 for its subscales, all indicative of good reliability.

A pilot study involving 50 nurses was conducted to test the tool’s feasibility and comprehensibility. The results indicated that the tool was well-received, with participants finding it clear, relevant, and easy to complete. Overall, the tool demonstrates strong face validity, reliability, and feasibility, suggesting its suitability in assessing moral sensitivity, personality traits, and safety attitudes in research contexts.

The researcher briefed the staff nurses on the study’s objectives. The researcher then visited the designated settings three days a week, on the morning and afternoon shifts. In front of the researcher, each nurse was asked to complete the self-administered questionnaire. The time required to complete the questionnaire varied between 20 and 30 min.

The researchers seek to control the threats of internal and external validity. Obtaining a sample of critical care nurses from different hospitals in the Sohag governorate, which makes the sample more representative, can mitigate the threat to external validity (Generalization). The selection of these hospitals was done by the director of the Sohag Health Directorate, which reduces threats to internal validity (Selection bias). Controlling the instrumentation threat to internal validity was done by using valid and reliable data collection tools.

### Statistical analysis

Statistical analyses were performed with IBM SPSS 26.0 software. The data were tested for normality using the Anderson-Darling test and for homogeneity variances. Categorical variables were described by number and percent (N %), whereas continuous variables were described by the mean and standard deviation (Mean, SD). A two-tailed *P* > .05 was considered statistically non-significant, *p* < .05 significant, *P* < .01 moderately significant, and *P* < .001 highly significant. Pearson correlation analysis was used to assess the inter-relationships between total scale scores. Finally, the PROCESS macro technique for SPSS was applied to examine the mediation model.

## Results

Table (1) shows that 55.2% of the studied nurses were aged between 40 and 50 years and older, with a mean age of 41.45 ± 6.41. 55.6% were female, 69.8% were married, and 40.9% had a baccalaureate degree in nursing. In addition, 57.8% of studied nurses worked in the ICU, and 50% had ≥ 10 years of working experience with a mean **±** SD 9.84 **±** 3.26, 84.5%. 71.6% of them did not have training courses related to safety attitudes.

Table (2) Presents the personality profile of the studied nurses, where the total mean score of personality traits was (150.012 **±** 9.628) with an average score (of 3.4094 **±** 0.21883). The higher mean scores of personality traits were at conscientiousness (34.59 ± 2.765) followed by openness (36.163 ± 4.620), then extraversion (25.887 ± 2.284) compared to the lowest mean scores that were related to agreeableness (27.86 ± 3.1039) and neuroticism (25.508 ± 2.628).

Table (3) Reveals descriptive statistics of moral sensitivity subscales, where the mean score for total moral sensitivity was (121.73 ± 13.861) with a moderate average score (4.5088 ± 0.51338). The highest mean scores of moral sensitivity subscales were for interpersonal orientation (22.76 ± 3.339) and moral meaning (26.97 ± 4.279). While the lowest mean scores were for expressing benevolence (16.39 ± 3.889) and modifying autonomy (24.33 ± 6.712).

Table (4) Displays that the mean score of total safety attitude was (73.254 ± 11.368) with a low average score (2.4418 ± 0.37894). The highest mean scores of safety attitude subscales were related to perceptions of management (10.413 ± 2.613), followed by working conditions. While the lowest mean scores were related to safety climate (15.620 ± 3.046) and stress recognition (11.47 ± 2.819).

Table (5) clarifies the Pearson analysis results of the correlation among personality traits, moral sensitivity, and safety attitude. Pearson analysis reported a positive correlation between personality traits, moral sensitivity, and safety attitude (*r* = .214**, *r* = .218**, & *r* = .365**), respectively.

**Fig. 2:**
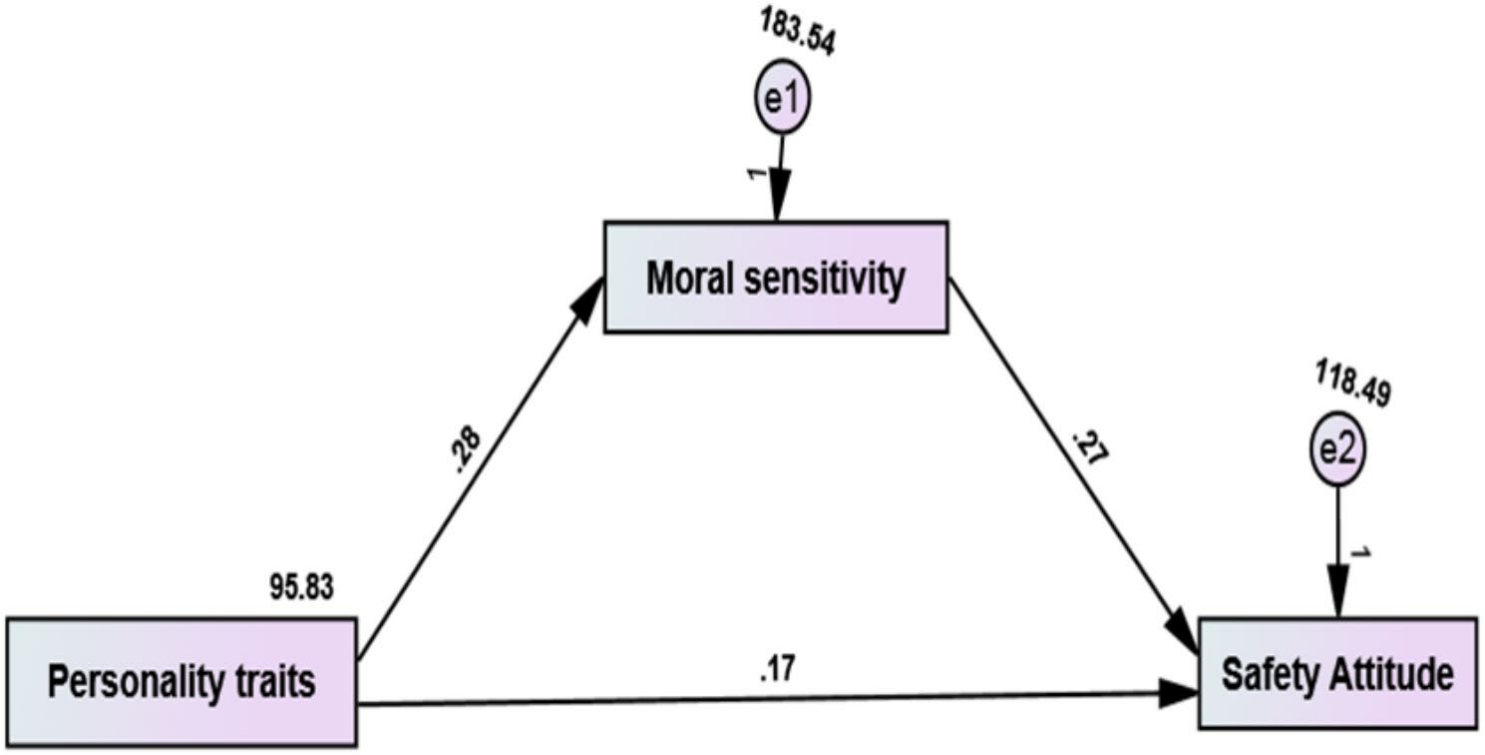
Mediation analysis model with Personality traits as the independent variable, moral sensitivity as the mediator, and safety attitude as the dependent variable. χ2 = 224.157; *P* = .001; χ2/df < 1.546; GFI = 0.940; RMSEA = 0.018; NNFI = 0.956; SRMR = 0.165; CFI = 0.970; AGFI = 0.921; IFI = 0.915 & TLI = 0.965, χ2 = Discrepancy Chi Square; χ2/df = Chi Square/Degrees of Freedom; CFI = Comparative Fit Index); IFI = Incremental Fit Index; RMSEA=, Root Mean Square of Error Approximation; NNFI = Non-Normed Fit Index; SRMR = Standardized Root Mean Square Residual; GFI = Goodness of Fit Index; AGFI = Adjusted Goodness of Fit; IFI = Incremental Fit Index& TLI = Tucker-Lewis Index.

Figure (2) and Table (6) depict the mediation analysis of moral sensitivity in relation to the relationship between personality traits and safety attitude. The pathway from personality traits to moral sensitivity (B = 0.285, *P* = .002) and the pathway from moral sensitivity to safety attitude (B = 0.272, *P* < .0001) was positive and statistically significant. Moreover, the pathway from explicit personality traits to a safety attitude is positive and significant (B = 0.168, *P* = .024). Also, Model Fit Coefficients parameters were (χ2 = 224.157; *P* = .001; χ2/df < 1.546; GFI = 0.940; RMSEA = 0.018; NNFI = 0.956; SRMR = 0.165; CFI = 0.970; AGFI = 0.921, IFI = 0.915 & TLI = 0.965). These results clarified the positive direct effect of moral sensitivity and personality traits on safety attitude while the positive indirect effect of personality traits on safety attitude.

**Table 1:** Distribution of studied nurses according to their personal characteristics (*n* = 232)

Personal Characteristics	Categories	No	(%)
**Age**	< 30 year	28	12.1
	30 < 40years	76	32.8
	40–50 years and more	128	55.2
	**Mean ±** SD	41.45 **±** 6.41	
**Gender**	Male	103	44.4
	Female	129	55.6
**Marital status**	Single	67	28.9
	Married	162	69.8
	Divorced	3	1.3
**Educational Qualification**	Nursing Diploma	41	17.7
	Technical institute	73	31.5
	Baccalaureate	95	40.9
	Specialized Diploma	13	5.6
	Post graduated education	10	4.3
**Department**	Intensive care units (ICU)	134	57.8
	Emergency departments	98	42.2
**Year of experience**	< 5	43	18.5
	5 < 10	73	31.5
	≥ 10	116	50.0
	**Mean ±** SD	9.84 **±** 3.26	
**Nurses position**	Staff nurses	196	84.5
	Head nurses	30	12.9
	Nurses’ supervisor	6	2.6
**Training courses related to safety attitude**	Yes	66	28.4
	No	166	71.6

**Table 2:** Descriptive analyses of the big five personality traits (*n* = 232)

Subscales	Item	Mean score	Average score	Rank
Conscientiousness	9	34.59 **±** 2.765	3.8434 **±** 0.30723	1
Openness	10	36.163 **±** 4.620	3.6164 **±** 0.46206	2
Extraversion	8	25.887 **±** 2.284	3.2360 **±** 0.28550	3
Neuroticism	8	25.508 **±** 2.628	3.1886 **±** 0.32856	4
Agreeableness	9	27.86 **±** 3.1039	3.0958 **±** 0.34488	5
Total	44	150.012 **±** 9.628	3.4094 **±** 0.21883	-

**Table 3:** Descriptive analyses of moral sensitivity subscales (*n* = 232)

Subscales	Item	Mean score	Average score	Rank
Interpersonal orientation	4	22.76 ± 3.339	5.6918 ± 0.83475	1
Moral meaning	5	26.97 ± 4.279	5.3948 ± 0.85583	2
Experiencing conflict	3	14.27 ± 3.255	4.7572 ± 1.08510	3
Reliance on medical authority	4	16.99 ± 3.544	4.2489 ± 0.88595	4
Expressing benevolence	4	16.39 ± 3.889	4.0981 ± 0.97219	5
Modifying autonomy	7	24.33 ± 6.712	3.4766 ± 0.95881	6
**Total**	27	121.73 ± 13.861	4.5088 ± 0.51338	

**Table 4:** Descriptive analyses of safety attitude subscales (*n* = 232)

Subscales	Item	Mean score	Average score	Rank
Perceptions of management	4	10.413 ± 2.613	2.6034 ± 0.65347	1
Working conditions	4	10.008 ± 2.843	2.5022 ± 0.71092	2
Teamwork climate	6	14.23 ± 2.836	2.3728 ± 0.47267	3
Job satisfaction	5	11.495 ± 3.711	2.2991 ± 0.74229	4
Safety climate	7	15.620 ± 3.046	2.2315 ± 0.43528	5
Stress recognition	4	11.47± 2.819	2.8696 ± 0.70479	6
**Total**	30	73.254 ± 11.368	2.4418 ± 0.37894	-

**Table 5:** Correlation between studied variables (*n* = 232)

Variables	Personality traits	Moral sensitivity	Safety attitude
Personality traits	1	0.214^**^	0.218^**^
Moral sensitivity	-	1	0.365^**^
Safety attitude	-	-	1

r = Pearson Correlation, **. Correlation is highly significant at the 0.01 level (2-tailed), *. Correlation is significant at the 0.05 level (2-tailed)

**Table 6:** Path analysis

Paths	Estimate (B)	S.E.	C.*R*.	t	*P*	Indirect Effect	Direct Effect
Moral sensitivity ← Personality traits	0.285	3.127	0.091	11.978	0.002**	0.000	0.285
Safety attitude ← Moral sensitivity	0.272	5.152	0.053	0.239	0.000***	0.000	0.272
Safety attitude ← Personality traits	0.168	2.253	0.075	0.519	0.024*	0.078	0.168

B: Unstandardized Coefficients; SE: standard error; C.R: Critical ratio; t: t-test of significance& * statistically significant at *p* ≤ .05

## Discussion

In the scientific literature on the issue, personality traits are considered a pattern of nurse behavior that is manifested and then practiced in everyday life. They affect patient safety attitudes as they are associated with how nurses’ moral sensitivity characteristics interfere with ethical patient issues and moral reasoning, which is an essential aspect for nurses every day and is affected by personality traits [31–32]. So, this study was designed to assess the role of moral sensitivity of critical care nurses on their safety attitude and personality traits.

Firstly, personality traits were examined based on the personality profile of the participants. The means score of total personality traits was neutral toward positive traits among participants. The highest mean scores were related to conscientiousness, followed by openness and extraversion, while the lowest mean score was related to agreeableness and neuroticism. This may be explained by a high level of conscientiousness among nurses in the critical care units or intensive care unit, which is related to their degree of commitment to their work and the amount of impulse control they possess related to the critical patient’s case in this unit.

This result is supported by many previous studies [31, 32, 33–34–35], which reported that the highest mean score was for conscientiousness and the lowest score was for neuroticism. According to John and Srivastava in 1999 [26], conscientious people are achievement-oriented, have good impulse control, are rule-conforming, and are consistent. Nurses in critical care units, mainly in the ICU, must have a high level of conscientiousness. That reflects their degree of commitment to their work and the amount of impulse control they possess related to critical patients.

Neurotic individuals have a predisposition to experiencing negative emotions, such as anxiety, angry hostility, depression, self-consciousness, impulsiveness, and vulnerability. Wang and colleagues (2017) indicated that nurses got the lowest score for neuroticism [36]. Besides that, a recent study in Japan supported the current study results that neuroticism hurt critical care nurses’ competencies [26].

Agreeableness is characterized by pleasantness and a desire to preserve interpersonal harmony [26]. A highly agreeable person is thought to be cooperative. The lowest mean score for agreeableness may be attributed to the critical care unit’s high workload and stress levels. The constant influx of diverse cases and unexpected changes in patient’s conditions leave little time or energy for nurses to offer support or assistance to their colleagues [37, 38].

A scoping review of dominant personality types among nurses and paramedics revealed that the prevalent traits among them are characterized by low neuroticism and higher extraversion [39]. In the current study, nurses were characterized as having low extroversion, which means they tend to spend time alone without social stimulation. This is congruent with a previous study finding that nurses need less social connection in the work environment related to high workload and no time to develop social relations with other team members (Li et al., 2023) [40]. 

Second, we examined nurses’ moral sensitivity based on our findings, clarifying that from total mean scores, the participants had an average level of moral sensitivity. The highest mean score of the moral sensitivity subscale was for interpersonal orientation and moral meaning. The lowest mean score was for modifying autonomy, expressing benevolence, reliance on medical authority, and experiencing conflict.

The highest mean score on the moral sensitivity subscale was observed in the areas of interpersonal orientation and moral meaning. This result implies that participants are particularly attuned to the interpersonal dynamics of ethical situations and place a high value on the moral significance of their actions. This orientation towards interpersonal relationships and moral significance aligns with the nature of nursing practice, which often involves complex ethical dilemmas and requires a deep understanding of the impact of one’s actions on others. Consistent with earlier research by Dalcali and Şendir in 2016 and Yildiz and Yildirim in 2023 [41–42], our study indicates that nurses exhibit moderate levels of moral sensitivity. This aligns with the findings of Arslan and Calpbinici in 2018 [43], who reported a similar mean score for moral sensitivity (95.89 ± 24.34) among pediatric nurses, suggesting a moderate level of moral sensitivity in this population.

Borhani, Abbaszadeh, and Hoseinabadi-Farahani in 2016 [44] identified that among nurses, the lowest mean scores were related to the dimensions expressing benevolence and autonomy. “Expressing benevolence” encompasses concepts such as honesty, trust in nurse-patient relationships, consideration of patient reactions to care, and patient knowledge about their illness. Similarly, Charles (2017) [45] found that nurses had low autonomy, which involves respecting patient autonomy and avoiding medical paternalism. This suggests that nurses may feel disempowered by medical paternalism or lack the skills needed for shared decision-making, potentially leading to increased patient dependency on passive relationships.

Third, we examined nurses’ safety attitudes based on our findings; the mean scores for total safety attitudes indicated that nurses’ safety attitudes were nearly neutral. This result is supported by a previous Egyptian study in 2017 [21]. Regarding the mean score for its subscales, the highest mean score was related to perceptions of management, working conditions, and teamwork climate; however, the lowest mean score was related to stress recognition and safety climate.

The highest mean score was observed in perceptions of management, indicating that participants generally perceive their management as supportive of safety practices. This positive perception of management is crucial, as leadership support is known to be a key factor in promoting a culture of safety and influencing frontline staff’s safety behaviors. Moreover, the high mean scores for teamwork climate and working conditions indicate that participants perceive a positive teamwork climate and favorable working conditions within their units [46–47].

The current study revealed that the lowest score was related to stress recognition. It can be reasoned that nurses need to have the needed skills to assess their own internal and external stressors and recognize the stress when it occurs. This is in line with those of Raftopoulos and Pavlakis (2013) and Henry, Hunt, Kroetch, and Yang (2012) [48–49] in that the lowest attitude score was in the domain of stress recognition. Besides that, the safety climate subscale was at a lower score. The safety climate is the perception of strong and constructive organizational commitment to safety. This result is consistent with a previous study by Gambashidze, Hammer, Ernstmann, & Manser in 2020 [50], which identified the safety climate as one of the weakest factors. This can be proved as the studied nurses did not receive any training courses related to safety, which reflected a negative organizational commitment to safety.

This result is supported by related studies [51]. Also, other studies [52–53] showed a moderate level of professional attitude regarding patient safety, which is lower than the international standards attitude regarding patients’ safety. In a previous study by Saberi et al. in 2017 [53] showed that the highest rate of positive attitudes toward the patient safety culture belonged to the dimension of ‘management perception’. Furthermore, Al-Mugheed et al., in 2022, indicated that overall patient safety attitudes among doctors and nurses were negative [54].

The current study’s findings align with research conducted among doctors and nurses in Saudi Arabia, revealing negative attitudes toward all safety domains, which pose challenges for developing a safety culture [55]. This is consistent with studies by Lee et al. in 2010 and Mahfoozpour et al. in 2012 [56–57], which reported a high percentage of positive attitudes among healthcare providers toward the perception of management. The positive perception in this dimension may be attributed to unit management rather than hospital management, indicating that unit management is more supportive of a patient safety culture, which ideally should be reflected throughout the entire hospital. The safety climate score of 17.49 (SD = 4.09) reflects the perception of a strong and constructive organizational commitment to safety [21].

Fourth, we examined the correlation between personality traits, moral sensitivity, and safety attitude; based on our findings, Pearson analysis reported a positive correlation.

This study’s findings are consistent with research conducted in the United Kingdom, which found that moral sensitivity, encompassing concepts such as authority, loyalty, sanctity, fairness, and care, is related to individual differences in personality traits [58]. Additionally, Jeong, Nam, Kim, and Son in 2021 [59] discovered a significant positive correlation between safety nursing activities and moral sensitivity, indicating that moral sensitivity positively influences safety nursing activities. These findings are in line with a previous Iranian study among nurses, which also showed a significant positive correlation between moral sensitivity, care behavior, and various dimensions [60]. Nurses’ moral sensitivity was observed alongside a notable degree of missed care, which poses risks to patient safety [61].

In a study conducted in Korea by Han, Seo, Kim, and Kim in 2018 [62] on moral sensitivity and compliance with infection standard precautions among 214 general hospital nurses, it was found that compliance with infection-related standard precautions increased significantly with higher levels of moral sensitivity. This suggests that nurses’ adherence to patient safety-related behavior is influenced by their internal sense of responsibility and ethics. Therefore, customized strategies to enhance moral sensitivity and promote quality safety nursing activities are essential.

Similarly, Mohammadi et al. in 2022 [63] highlighted the significance of moral sensitivity in ensuring ethical decisions and safe care, particularly in special care units where critical patients are treated. The authors suggested that moral sensitivity plays a crucial role in providing safe and ethical care in challenging situations.

In the broader literature, moral sensitivity is consistently identified as a critical attribute for nurses, enabling them to deliver high-quality and safe care, especially in critical patient care settings where they frequently encounter ethical and moral dilemmas. The ability to identify and respond to these challenges is essential, as moral insensitivity or an inability to recognize ethical issues may lead to inappropriate care behavior. Nurses’ decisions and actions are heavily influenced by their level of moral sensitivity, making it a key factor in ensuring responsive and ethical care delivery, particularly in complex healthcare environments [64].

Finally, the findings demonstrated that moral sensitivity acted as a mediating factor between personality traits and safety attitude. These findings confirm the positive direct effects of moral sensitivity and personality traits on safety attitude, as well as the positive direct effects of personality traits on moral sensitivity.

However, we could not directly compare our findings to the literature due to the rarity of studies that examined the mediating effects of moral sensitivity on the relationship between personality traits and safety attitude. According to Rezapour-Mirsaleh et al. in 2022 [65], personality traits such as conscientiousness, empathy, and mindfulness have a direct effect on moral sensitivity, and nurses who possess enough moral sensitivity could create an atmosphere in which patients not only have their rights respected but also feel safe in these conditions, and the goals of healthcare can be accomplished [21].

Any moral or clinical decision-making in nursing is directly influenced by nurses’ personality traits that involve cognitive processes and depend on the way nurses estimate the importance of received messages, their priorities, and their capability of recognizing and responding to the often ambiguous clinical and nonclinical scenarios in the workplace [66–67].

According to Dalla., Zoboli., & Vieira, in 2016 [68], moral sensitivity is not just an issue of feeling, that is, trusting your own emotions for identifying a moral conflict, but it is related to– a personality component acquired through each one’s experience, which allows one to perceive the moral meaning of a specified situation. Moral sensitivity may be understood as a type of emotional response, being a personal component, which is necessary when one deals with ethical problems. In addition, the moral sensitivity of nurses is correlated with a higher level of compliance with standard precautions, which is a safety nursing protocol [69], and this leads to increased nurses’ attention to ethical considerations in the quality of care [70].

Critical care nurses in emergency and intensive care unit care for patients generally attend to the emergency unit due to acute diseases or injuries that require rapid treatment. Thus, nurses working in the emergency unit are occasionally obliged to initiate immediate treatment without obtaining a detailed history from patients [71]. Emergency unit nurses experience problems, especially when the patient is unconscious due to not being able to obtain sufficient information on the patient’s medical condition, so it is very vital to have positive personality traits with high moral sensitivity [72] because moral sensitivity has a positive effect on safety nursing activities [62, 73].

### Limitation

Convenient sampling involves selecting participants who are readily available and accessible, which may introduce bias into the study. In this case, the study focuses solely on critical care nurses, limiting the generalizability of the findings to other nursing specialties or healthcare settings.

Furthermore, by exclusively studying critical care nurses, the study may overlook important differences in moral sensitivity, safety attitudes, and personality traits among nurses in different specialties. Critical care nurses may have unique job demands and experiences that could influence their moral sensitivity and attitudes toward safety differently from nurses in other specialties.

## Conclusion

The study found that more than half of the participants have positive personality traits; also, higher mean scores were in conscientiousness followed by openness and extraversion compared to the lowest mean score related to agreeableness and neuroticism. Studied nurses had average levels of moral sensitivity; the highest mean score of moral sensitivity subscale was on interpersonal orientation and moral meaning, while the lowest mean score was for modifying autonomy and reliance on medical authority, expressing benevolence. Nearly half of the participants’ nurses have a positive safety attitude. Regarding safety attitude subscales, the highest mean score was related to perceptions of management, while the lowest mean score was related to safety climate and job satisfaction. There was a positive correlation between personality traits, moral sensitivity, and safety attitude. Finally, the findings demonstrated that moral sensitivity acted as a mediating factor between personality traits and safety attitude.

### Implications

This study has significant implications for nursing practice and management. It underscores the importance of integrating personality assessment and moral sensitivity training into nursing education and professional development programs. By enhancing nurses’ self-awareness and sensitivity to ethical dilemmas, institutions can improve safety attitudes and, consequently, patient care outcomes.

Secondly, the identification of moral sensitivity as a mediating factor suggests that interventions targeting moral development could positively influence safety attitudes among critical care nurses. Programs aimed at enhancing moral sensitivity, such as ethics training and reflective practices, may lead to more conscientious decision-making regarding patient safety. Additionally, fostering a supportive work environment that values ethical conduct and encourages open dialogue about moral issues could further enhance the impact of such interventions.

Longitudinal studies tracking the development of these constructs over time could provide a more comprehensive understanding of their dynamics and help identify effective strategies for promoting ethical practice and patient safety. Furthermore, investigating the role of contextual factors, such as organizational culture and leadership styles, in influencing these relationships could provide additional avenues for improving safety attitudes and ethical decision-making in critical care nursing.

## Electronic supplementary material

Below is the link to the electronic supplementary material.

Supplementary Material 1

## Data Availability

The datasets used and/or analysed during the current study available from the corresponding author on reasonable request.

## Abbreviations

MSQ: Moral sensitivity questionnaire
SAQ: Safety attitudes questionnaire
CNI: Consequence sensitivity (C), norm sensitivity (N), and generalized inaction versus action preferences
ICU: Intensive care unit
ED: Emergency departments
