# Supplementary material for: Revelation of the mediation role of moral sensitivity on safety attitude and personality traits among critical care nurses
Source: BMC Nurs. 2025 Mar 8;24:261. doi: 10.1186/s12912-025-02868-6 (PMC11889890; doi:10.1186/s12912-025-02868-6)
Supplement: Supplementary file 1 — Supplementary Material 1 [file 12912_2025_2868_MOESM1_ESM.docx]

**Tool I: Personal data sheet**

| **Personal Characteristics** | **Categories** |
| --- | --- |
| **Age** | **<30 year** |
|  | **30 – 40years** |
|  | **40 - > 50 years** |
| **Gender** | **Male** |
|  | **Female** |
| **Marital status** | **Single** |
|  | **Married** |
|  | **Divorced** |
| **Educational Qualification** | **Nursing Diploma** |
|  | **Technical institute** |
|  | **Baccalaureate** |
|  | **Specialized Diploma** |
|  | **Post graduated education** |
| **Department** | **ICU** |
|  | **Emergency** |
| **Year of experience** | **<5** |
|  | **5<10** |
|  | **≥ 10** |
| **Nurses position** | **Staff nurses** |
|  | **Head nurses** |
|  | **Nurses’ supervisor** |
| **Training courses related to safety attitude** | **Yes** |
|  | **No** |
